# Anti-Hyperglycemic and Anticholinergic Effects of Natural Antioxidant Contents in Edible Flowers

**DOI:** 10.3390/antiox8080308

**Published:** 2019-08-15

**Authors:** Paulina Nowicka, Aneta Wojdyło

**Affiliations:** Department of Fruit, Vegetable and Plant Nutraceutical Technology, Wrocław University of Environmental and Life Sciences, 37 Chełmońskiego Street, 51-630 Wroclaw, Poland

**Keywords:** edible flowers, bioactive compounds, anti-diabetic activities, anti-aging activities

## Abstract

In this study, 16 selected edible flowers were evaluated for their content of bioactive compounds (polyphenols, carotenoids, triterpenoids) and for their anti-diabetic (ability to inhibit α-amylase and α-glucosidases) and anti-aging (ability to inhibit acetylcholinesterase and butyrylcholinesterase) activities. Most of the flowers analyzed in this study have not been examined in this respect until now. Contents of the analyzed bioactive compounds differed significantly among the flowers. In particular, the highest contents of carotenoids and triterpenoids were determined in marigold, arnica, lavender, and daisy; in turn, the highest contents of phenolic acids, procyanidin polymers, and total polyphenols were assayed in hawthorn, primrose, and linden blossom. There was a positive correlation between the content of isoprenoids in edible flowers and their anti-aging activity, and between the content of polymeric procyanidins and flowers’ ability to inhibit α-glucosidase. In conclusion, edible flowers may be used to produce functional foods as well as for medical purposes.

## 1. Introduction

According to estimates by the World Health Organization (WHO), chronic non-communicable diseases like cardiovascular diseases, neoplasms, obesity or diabetes, are today the main causes (more than 75%) of death worldwide. One of the ways of their effective prophylaxis is implementation of a diversified and well-balanced diet in which plants play a key role. Increasing awareness of consumers in this area and, simultaneously, a growing interest of food producers in this subject have initiated a search for raw materials with a high nutritive value and attractive sensory traits. Investigations conducted so far, related to the global search for nutraceuticals in plants, have focused mainly on bioactive compound profiles and on the health potential of fruits, vegetables, herbs, and cereals. Recent focus has also shifted to edible flowers, which until now have largely been perceived as decorative elements only. Edible flowers add a fresh and exotic aroma, a delicate flavor, and a visual appeal to foods, characteristics which make them increasingly used in gourmet cuisine. In addition, they provide multiple health benefits which have been exploited for years, especially in folk medicine in China [1,2]. Generally, the chemical composition of edible flowers is similar to that of other edible plants. They have high contents of water, vitamins, different carbohydrates, and minerals [3,4]. They are also a good source of phytochemicals, including phenolic compounds [4,5].

Phenolic compounds constitute a large group of natural organic substances that occur in various morphological parts of plants. They exhibit especially strong antioxidative properties that protect defense systems of the body against destructive effects of free radicals [6]. Therefore, their intake prevents the disruption of the antioxidative equilibrium and by this means prevents the incidence of many disease entities, e.g., neoplasmic diseases, cardiovascular diseases, diabetes, or neurodegenerative diseases [7,8,9,10,11]. Another group of secondary metabolites of plants that exhibit health-promoting properties are isoprenoids which include triterpenes, carotenoids, and chlorophylls. Similar to polyphenols, carotenoids are classified as both preventive and intervention antioxidants, and offer valuable biological properties, the best documented of which is their provitamin activity [12,13]. Apart from carotenoids, chlorophylls are also valuable dietary constituents. Being natural plant pigments, they occur primarily in leaves and green parts of plants. They exhibit anti-inflammatory, anti-carcinogenic, and antioxidative properties. The consumption of carotenoid-rich plants lowers arterial blood pressure, stimulates gut peristalsis, and prevents anemia [14,15,16]. Another group of phytocompounds with documented health-promoting effects includes triterpenes. Plants with high contents of pentacyclic triterpenes are commonly used in the phytotherapy owing to their valuable therapeutic properties, i.e., antiviral, antioxidative, anti-inflammatory, anti-carcinogenic, and cytoprotective ones [17,18].

Flowers may contain many of these natural antioxidants, including polyphenols and isoprenoids, but this has not been confirmed explicitly and requires careful analysis. Among the rich world of edible flowers, only a few have been tested. The content of polyphenols and antioxidative activity of calendula, rose, hibiscus, jasmine, or lavender samples have already been studied [3,4,5,19]. However, little is known about derivatives of flavonols and flavan-3-ols, including procyanidin polymers, in edible flowers. In addition, sparse literature data are available regarding contents of triterpenes in flowers, which seem to be extremely important considering their bioactive properties. To the best of authors knowledge, there are also no reports on the bioactive properties of flowers, including their anti-hypoglycemic and anticholinergic effects. Therefore, the main aim of this study was to determine contents of bioactive compounds (polyphenols, carotenoids, chlorophylls, and triterpenoids) and health-promoting properties (anti-hypoglycemic and anti-aging) of 16 selected species of edible flowers. These studies allow the determination of new directions for the use of edible flowers, including in the food industry for the preparation of novel food with pro-health properties.

## 2. Materials and Methods

### 2.1. Plant Materials

All edible flowers (Table 1) were purchased in commerce (Wrocław, Poland). The fresh flowers were cleaned, freeze-dried and ground into a fine powder by laboratory mill.

### 2.2. Determination of Polyphenolic Compounds, Including Polymeric Procyanidins

The quantitative analysis of polyphenolic compounds by Ultra-Performance Liquid Chromatography (UPLC) was performed according to the protocol described by Wojdyło et al. [20]. The freeze-dried powder of edible flowers (∼0.5 g) was vortexed for 1 min with 5 mL methanol/water/acetic acid/ascorbic acid (30:68:1:1, v/v) and sonicated for 20 min. All supernatant was collected after being centrifuged, filtered by 0.20 μm hydrophilic membrane and finally used for phenolic compound quantification by Ultra-Performance Liquid Chromatography Photodiode Array Detector (UPLC-PDA). In turn, the content of polymeric procyanidins was performed by the phloroglucinol method previously described by Kennedy et al. [21]. All measurements were repeated three times. The results were expressed as mg/100 g dry matter (dm) of edible flowers.

### 2.3. Determination of Carotenoids and Chlorophylls by UPLC Method

The quantitative and qualitative analysis of carotenoids and chlorophylls by UPLC was performed according to the protocol described earlier by Wojdyło et al. [20]. The freeze-dried powders of edible flowers (0.15 g) containing 10% MgCO_3_ and 1% BHT were shaken with 5 mL of a mixture consisting of methanol/acetone/hexane (1:1:2, v/v/v) for 30 min in the dark. Obtained supernatants (after the 3–4 times re-extraction of solid residue) were collected after being centrifuged and then were evaporated to dryness. The pellet was diluted using from 1 to 2 mL of 100% methanol, filtered, and used for analysis by UPLC. All measurements were repeated three times. The results were expressed as mg/100 g dm of edible flowers.

### 2.4. Determination of Triterpenoids by UPLC Method

For the extraction and determination of triterpenoids, a protocol similar to that described earlier by Kolniak-Ostek [22] was followed. The freeze-dried powder of edible flowers (0.2 g) was extracted with 5 mL of ethyl acetate and 5 mL of hexane and sonicated for 15 min with occasional shaking. After the first extraction, the samples were kept at 4 °C overnight and on the next day the samples were re-extracted in the same conditions. The slurry was centrifuged, then the supernatant was evaporated to dryness and finally re-extracted using 2 mL of 100% methanol. The extract prepared in this way was filtered and used for analysis. All determinations were repeated three times and the obtained results were expressed as mg/100 g dm of edible flowers.

### 2.5. Determination of Anti-Hyperglycemic and Anticholinergic Activities

Both for determination of anti-hyperglycemic and anticholinergic activities were used the same plant’s extracts. The freeze-dried powders of edible flowers (∼0.5 g) were mixed with 5 mL of methanol:water (80:20%, v/v) with addition of 1% HCl, sonicated at 20 °C for 15 min and left for 24 h at 4 °C. Then the extracts were again sonicated for 15 min, and centrifuged. The obtained supernatants were used for analysis.

The α-amylase and α-glucosidase inhibitory effect of the edible flowers extracts was assayed according to the procedure described previously by Nowicka, et al. [23]. The inhibition of α-amylase activity was determined by measuring the reducing groups released from starch. In turn, the α-glucosidase activity was determined by measuring the amount of glucose hydrolyzed from *p*-nitrophenyla-α-d-glucopyranoside. Acarbose was included as a positive control. The results were read at 540 nm (α-amylase) and 405 nm (α-glucosidase).

Acetylcholinesterase (AChE) and butyrylcholinesterase (BuChE) activity was determined by the Ellman’s method with slight modifications presented by Jin et al. [24]. The AChE inhibition assay was performed using 20 μL of substrate (2.5 mM acetylcholine iodine), 100 μL DTNB (1 mM), and 20 μL of enzyme (acetylcholinesterase—1 U/mL). In turn, the measuring of the BuChE inhibition effect was performed using 20 μL of butylcholine chloride (2.5 mM), 100 uL DTNB (1 mM), and 20 μL of enzyme (butyrylcholinesterase—1 U/mL). For both methods the absorbance was measured at 405 nm.

The enzyme inhibition assays were expressed as IC_50_ value (mg/mL). IC_50_ expressed in mg/mL is a quantitative measure that indicates how high concentration of edible flowers (mg/mL) is needed to inhibit, in vitro, a given solution of biological component by 50% (1 U/mL).

### 2.6. Statistical Analysis

Statistical analyses, i.e., significant differences (*p* ≤ 0.05) between mean values (*n* = 3) by one-way ANOVA, Duncan’s multiple range test, and principal component analysis (PCA), were performed using Statistica version 12.5 (StatSoft, Krakow, Poland).

## 3. Results and Discussion

### 3.1. Content of Polyphenolic Compounds in Selected Edible Flowers

The main fractions of phenolic compounds identified in the edible flowers and their contents are presented in Table 1. Contents of total polyphenolics in the tested materials varied from 284 mg (cornflower) to 7108 mg (hawthorn) in 100 g dm. In general, the examined flowers can be divided into four basic groups in terms of the content of polyphenolic compounds. The first one includes edible flowers in which the total polyphenolic compounds account for over 2000 mg/100 g dm (hawthorn, primrose, kidney vetch, and linden blossom). Their characteristic feature is a very high content of polymeric procyanidins, ranging from 59% to 94% of the total polyphenols. The second group includes flowers with contents of total polyphenolics ranging from 1000 to 2000 mg/100 g dm, i.e., acacia, arnica, lavender, daisy, and black hollyhock. In turn, chamomile, mallow, and white dead-nettle constitute the third group with polyphenol content between 500 and 1000 mg/100 g dm. Elderberry, mullein, and cornflower—representing the fourth group—had the lowest content of polyphenols, i.e., <500 mg/100 g dm. The content of polyphenolic compounds determined in hawthorn was similar to that determined in black tea (*Camellia sinensis* L. 60 mg CE/g), whereas linden blossom contained similar amounts of polyphenols as *Osmanthus fragrans* L. (47 mg CE/g) [25]. In general, the conducted study showed significant differences in the contents of total polyphenolic compounds in the tested flowers. The same observations were made by other authors, who analyzed both edible flowers and herbs and indicated these differences to be caused by the botanical origin, genotypic and environmental differences within species, choice of plant parts tested, time of sample collection, and analytical methods [1,25,26,27]. Anthocyanins were identified in only six of the analyzed flowers, these being: black hollyhock (286 mg/100g dm), white dead-nettle (248 mg/100 dm), mallow (13 mg/100 g dm), lavender, cornflower, and mullein. Anthocyanin content in the latter three flowers was lower than 10 mg/100 g dm. In the case of black hollyhock and white dead-nettle, anthocyanins constituted 28% and 25% of the total phenolics, respectively. It was observed that the samples containing anthocyanins did not contain polymeric procyanidins in their composition, whereas the samples rich in the polymerized forms did not contain anthocyanins. Anthocyanins are natural pigments in plants, therefore they are present in flowers with colorful petals. The less brightly colored flowers (white with a little violet—cornflower or mullein) had only low amounts of anthocyanins. In turn, the flowers with pale petals (hawthorn, kidney vetch, linden blossom) have no anthocyanins, but high contents of polymeric procyanidins. This was also confirmed elsewhere [28,29].

In the group of flavonoids, we identified also flavonols, contents of which ranged from 11 mg to 850 mg per 100 g of dried samples, and flavan-3-ols with contents ranging from 132 to 1795 mg/100 g dm. The richest in flavonols were primrose (850 mg/100 g dm); acacia and marigold (330 and 337 mg/100 g dm, respectively); hawthorn (194 mg/100 g dm); black hollyhock and chamomile (132 and 113 mg/100 g dm, respectively). In turn, the highest contents of flavan-3-ols were determined in marigold (1795 mg/100 g dm); arnica and hawthorn (1397 and 1384 mg/100 g dm, respectively); lavender (989 mg/100 g dm); and primrose (926 mg/100 g dm). Other authors reported quercetin, kaempferol, myricetin and rutin (flavonols); apigenin and luteolin (flavones); and catechins and epicatechins (flavan-3-ols) to be the most common compounds in edible flowers [4,5].

Phenolic acid derivatives represent the next class found in the edible flowers. All of the analyzed samples contained phenolic acids, but their contents differed significantly and ranged from 1 mg/100 g dm in kidney vetch to 350 mg/100 g dm in daisy. The conducted study showed that among all 16 selected species, the flowers from the *Asteraceae* family were characterized by the highest content of phenolic acids, except marigold (15 mg/100 g dm). In the flowers from this family phenolic acids represented 26% (chamomile), 25% (daisy), 17% (cornflower), and 13% (arnica) of the total phenolics. Also Wojdyło et al. [30], who examined different herbs, reported high contents of phenolic acids (over 70% of the total polyphenols) in *Achillea millefolium* and *Echinacea purpurea* (*Asteraceae* family). Among the phenolic acids there are two sub-groups, i.e., hydroxybenzoic and hydroxycinnamic acids. According to authors who analyzed edible flowers, the most common hydroxybenzoic acids in flowers are vanillic and protocatechuic acids; in turn the most common hydroxycinnamic acids are chlorogenic, syringic, caffeic, ferulic, and p-coumaric acids [4,5].

### 3.2. Quantification of Isoprenoids in Selected Edible Flowers

Isoprenoids are a very large group of compounds which comprises: monoterpenes, triterpenes, and tetraterpenes (carotenoids and chlorophylls). Because of their high biological activity, they are widely used in the cosmetic and pharmaceutical industries. Although edible flowers have been sparsely investigated for contents of these compounds (isoprenoid profiles of edible flowers are known for species of the *Asteraceae* family, mainly marigold) [31,32], they are speculated to be rich sources of isoprenoids, which was verified in this study. 

Table 2 shows contents of carotenoids, chlorophylls, and triterpenoids in 16 selected edible flowers. The highest contents of carotenoids among the tested samples were found in marigold (721 mg/100 g dm), arnica (559 mg/100 g dm), and hawthorn (473 mg/100 g dm). The lowest carotenoid contents were detected in cornflower, acacia, elderberry, and primrose (30, 31, 33, and 34 mg/100 g dm, respectively). Other authors have indicated that the main carotenoid compound in edible flowers is lutein, which is responsible for their yellow pigment [29,33]. This may explain the very high content of carotenoids in marigold flowers, which have a strong orange color. Some authors also showed zeaxanthin and β-carotene among the predominating compounds [29]. Differences in carotenoid contents are consistent with other reports [29,33] which suggest they depend on the botanical origin, environmental differences within species, choice of plant parts tested, or growth conditions. In general, it has been shown that edible flowers are very rich sources of carotenoids. The analyzed samples contained much more of these compounds than the raw materials considered to be their rich sources such as peach (~242 mg/100 g dm), goji fruits (~213 mg/100 g dm), pear (~20 mg/100 g dm), or carrots (from 6 to 13 mg/100 g fm) [20,34,35]. Carotenoids exhibit various physiological activities. The consumption of products rich in carotenoids, including edible flowers (fresh form or infusions), alleviates symptoms of diabetic retinopathy, enhances glutathione peroxidation, and reduces blood level of the low-density lipoprotein (LDL) cholesterol fraction, which ultimately contributes to the effective prophylaxis of chronic non-communicable diseases [29,34].

Apart from carotenoids, chlorophylls were also evaluated in this study. They were detected only in 10 of the analyzed edible flowers, probably because these natural plant pigments occur primarily in leaves and green parts of plants, and not in their flowers. The highest contents of these compounds were measured in lavender (59 mg/100 g dm), arnica (38 mg/100 g dm), and mallow (29 mg/100 g dm). As in the case of carotenoids, synthesis of chlorophylls depends on the botanical origin, maturation stage, and variety of plants as well as on light conditions. In the growing period under long light exposure, chlorophylls are degraded to colorless catabolites, and carotenoids become perceptible [36,37]. Hence in flowers, the content of carotenoids is much higher than that of the green pigments.

Triterpenoids represent another major group of isoprenoids detected in the edible flowers. All of the analyzed samples contained triterpenoids, but significant (*p* ≤ 0.05) differences were observed among selected plants. Their highest content was determined in marigold (0.72 mg/100 g dm), while the lowest content was observed in elderberry, acacia, primrose, and cornflower (0.03 mg/100 g dm). In addition, their contents were strongly positively correlated (PC = 1.000) with contents of carotenoids. Definitely, a lesser dependence was observed between triterpenoids and chlorophylls (PC = 0.354). There is little literature data on triterpenoids in edible flowers. The available works refer only to their contents in plants from the *Asteraceae* family, especially *Chrysanthemum morifolium* and marigold. The predominant components of the triterpenoids identified in *Asteraceae* flowers are oleanane, taraxane, ursane, lupane, dammarane, cycloartane, and tirucallane [31,38]. Recent studies have reported that these compounds isolated from edible flowers exhibit anti-inflammatory effects; and are competitive and non-competitive inhibitors of serine proteases (trypsin and chymotrypsin) [38,39]. This suggests a possible mechanism of the plant defensive system under in vivo conditions and the bioactivity of these compounds. The presence of triterpenoids is not restricted only to *Asteraceae* flowers. Thus, it seems worth analyzing the quantitative and qualitative profiles of other edible flowers in the future.

### 3.3. Anti-Hyperglycemic and Anti-Aging Properties of Edible Flowers

In this study, we investigated also the inhibition of α-glucosidase, α-amylase, acetylcholinesterase (AChE), and butyrylcholinesterase (BuChE). Extracts of all of the selected edible flowers were tested for their inhibitory effect at different concentrations, which enabled calculating their IC_50_ values and establishing their biological activity. Respective results are provided in Table 3.

In humans, dietary carbohydrates are hydrolyzed by pancreatic α-amylase and intestinal α-glucosidase into monosaccharides which are suitable for absorption. Inhibition of these enzymes is one of the strategies to counteract metabolic abnormalities related to hyperglycemia and type 2 diabetes, and therefore the use of phytoextracts such as α-amylase and α-glucosidase inhibitors may represent an alternative approach in preventing diabetes mellitus. Significant (*p* ≤ 0.05) differences were found among the analyzed flowers with reference to their inhibitory activities toward α-amylase and α-glucosidase. The inhibition of α-amylase, presented as the IC_50_ values, ranged from 3.50 to 11.69 mg/mL. Among the 16 tested samples, kidney vetch and arnica (flowers with yellow color of petals) were the most active, while the white flowers (primrose and hawthorn) showed the weakest anti-hyperglycemic potential. The selected edible flowers showed lower inhibitory activities toward α-glucosidase than toward α-amylase. The IC_50_ values for α-glucosidase were from 10.18 mg/mL to >100 mg/mL. The most active flowers were primrose, hawthorn, linden blossom, and arnica. The determined inhibitory activity of edible flowers toward α-amylase is similar to that measured for the most active fruits, e.g., chokeberry (1.18 mg/mL), black currant (3.88 mg/mL) or cranberry (7.99 mg/mL). In the case of α-glucosidase, activities of edible flowers are similar to those of sweet cherry (91.51 mg/mL), bilberry (75.76 mg/mL) or black currant (85.79 mg/mL) [40]. As expected, acarbose showed the lowest IC_50_, establishing its relative potency as a glucosidase inhibitor. The acarbose extract also was a strong inhibitor of α-amylase, exhibiting an IC_50_ that was slightly lower than that of edible flowers. These data indicate that edible flowers extracts are similarly efficient as the drug acarbose in inhibiting α-amylase activity, but much weaker in case of α-glucosidase inhibition. It is well known that polyphenols can bind proteins through hydrogen bonding or hydrophobic effects, leading to their complexation and precipitation. This means that extracts of different plants could potentially inhibit enzymes by aggregation. The hypoglycemic effect of polyphenolic compounds results also from their antioxidative potential involved in restoring the insulin-secreting machinery in pancreatic cells, or their abilities to inhibit the activity of carbohydrate-hydrolyzing enzymes (α-amylase and α-glucosidase) [40,41,42]. In this study, a positive correlation was observed between the inhibitory activity toward α-glucosidase and the total content of polyphenols (PC = 0.655), content of polymeric procyanidins (PC = 0.473) and flavonols (PC = 0.394). In turn, the α-amylase inhibitory effect of edible flowers was positively correlated with anthocyanin content (PC = 0.260). Other authors suggested that anthocyanin-rich plant extracts were effective inhibitors of α-amylase [40,41]. Probably, this is due to the fact that the glycosylated anthocyanins may act as substrate mimics and competitively interfere with hydrolysis of the substrate. Furthermore, some researchers suggested that flavonols can interact with anthocyanins or hydroxycinnamic acids, thereby increasing the inhibition of α-glucosidase [40].

Alzheimer’s disease (AD) is an age-related neurodegenerative disorder characterized by progressive cognitive dysfunction leading to dementia. Treatment of AD symptoms relies primarily on the inhibition of two main forms of cholinesterase (AChE and BuChE) by ensuring adequate levels of acetylcholine (ACh) at neurotransmission sites [24]. Considering the fact that the inhibition of both enzymes is currently the most established approach to treat AD, in this study we determined both AChE and BuChE inhibiting effects of 16 edible flowers (Table 3). Little literature data can be found on the anti-aging activities of edible flowers. The available works address only the inhibitory effect of lavender, *Calendula arvensis*, and marigold toward AChE [43,44]. Therefore, the presented study is the first to examine the inhibitory activity of different species of edible flowers toward both AChE and BuChE. Significant (*p* ≤ 0.05) differences were found among the analyzed samples in their inhibitory potential against AChE and BuChE. The inhibition of AChE, presented as the IC_50_ value, ranged from 31.64 mg/mL (cornflower) to 191.63 mg/mL (mallow), while the inhibition of BuChE ranged from 51.81 mg/mL to 379.31 mg/mL. It was observed that edible flowers were stronger inhibitors against AChE than BuChE. The inhibiting potential of the analyzed samples against AChE was similar to that measured by Ercetin et al. (2012) in *Calendula arvensis* (31.24 mg/mL) and marigold (74.27 mg/mL), but poorer than in plants of the *Lamiaceae* family, including lavender [44]. It has been shown that the inhibitory effectiveness clearly depends on botanical origin of plants. The authors who investigated the anti-aging properties of plants indicate that there is a positive correlation between the content of hydroxycinnamic acids and triterpenoids. At the same time, they showed that single compounds operated much less than the complexes of bioactive compounds, which is an indicative of a possible synergistic interaction [43,44]. The presented study confirms positive correlations between the inhibitory effect of edible flowers toward ACh and content of triterpenoids, carotenoids (PC = 0.216), and total content of flavan-3-ols (PC = 0.194), but such a correlation was not detected in the case of phenolic acids. The analysis of the cholinesterase-inhibiting activities of edible flowers was a very important element of this study, because it showed that the compounds detected in the analyzed samples can be a healthy alternative to strongly addictive drugs currently dedicated to patients with senile dementia problems.

A healthy form of edible flowers, easily accessible to everyone may be infusions of edible flowers, drunk daily. In addition, an interesting direction of processing the edible flowers may also be encapsulation and the production of powders, which could be applied to smoothies or juices and drinks as an additive shaping the taste and pro-health properties of the finished products. Therefore, we are going to continue the study about the health-promoting properties of edible flowers, their formulations, bioaccessibility, and efficacy.

### 3.4. Principal Component Analysis (PCA)

**Figure 1:**
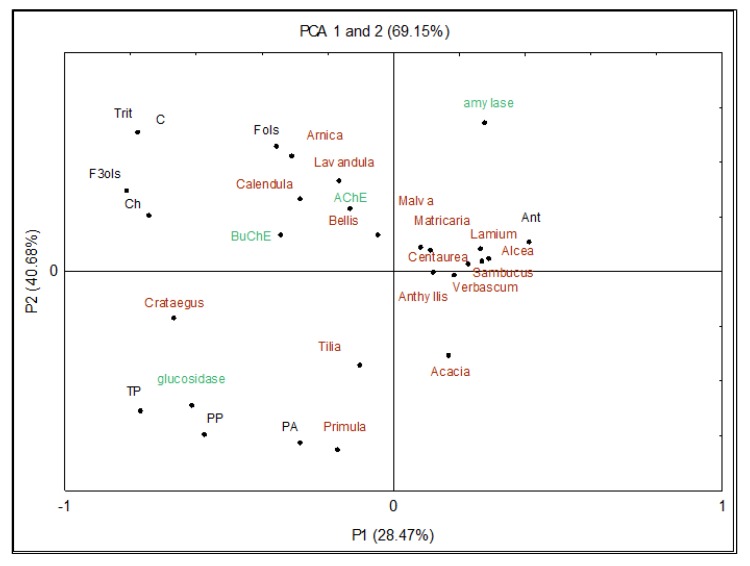
Principal Component Analysis (PCA) scores plot showing the relationship among bioactive compounds and biological activity of selected edible flowers. Tri—triterpenoids; C—carotenoids; F3ols—flavan-3-ols (monomers and dimers); Ch—chlorophylls; Fols—flavonols; Ant—anthocyanins; TP—total polyphenols; PP—polymeric procyanidins; PA—phenolic acids.

The PCA was conducted to better understand trends, confirm relationships between the 16 selected edible flowers, and to identify any group patterns (Figure 1). After the statistical analysis of all obtained results, the PCA model retained two principal components (PC), which explained 69% of the total variability (28% and 41% for PC1 and PC2, respectively). The PC1 was clearly identified with health-promoting properties (anti-hyperglycemic and anti-aging activities), while the PC2 was related to the content of bioactive compounds (polyphenols and isoprenoids).

Thus, it was shown that a common feature of marigold, arnica, lavender, and daisy was a high content of triterpenoids, carotenoids, and flavonols, which was strongly correlated with these flowers’ ability to inhibit AChE and BuChE. The PCA plots showed also that the primrose, hawthorn, and linden blossom were located close to α-glucosidase inhibitory activity, and that this property was positively correlated with contents of total polyphenols, polymeric procyanidins, and phenolic acids. In addition, PC2 arranged the samples according to their high content of anthocyanins (cornflower, mallow, white dead-nettle, black hollyhock), revealing simultaneously a strong relationship between the red color of petals and α-amylase inhibitory activity of the flowers.

## 4. Conclusions

This study demonstrated significant differences in both the content of bioactive compounds and health-promoting properties of 16 selected edible flowers. Some of them were characterized by a high content of highly biologically active polymeric procyanidins, i.e., hawthorn, primrose, and *Tilia cordata*. It has been shown that these compounds represent over 50% of the total polyphenols in these flowers, but also that their contents are positively correlated with α-glucosidase inhibitory effect. Other flowers, i.e., marigold, arnica, lavender, and daisy, can be used as healthy alternative to strongly addictive drugs currently dedicated to patients with senile dementia problems. They are also characterized by a high content of carotenoids and triterpenoids. In turn, mallow, *Lamium album*, and black hollyhock—flowers with high contents of anthocyanins—exhibit anti-hyperglycemic properties.

Generally, the edible flowers are attractive plants, containing many bioactive compounds with a high health-promoting potential. They can be a valuable source of bioactive compounds for the pharmaceutical industry in the production of dietary supplements to support the prevention and treatment of chronic non-communicable diseases.

## Figures and Tables

**Table 1:** Content of phenolic compounds (mg/100 g dm) in 16 selected edible flowers.

Kind of Edible Flowers Scientific Name (Common Name)	Family	Anthocyanins	Phenolic Acids	Flavonols	Flavan-3-ols (Monomers & Dimers)	Polymeric Procyanidins	Total Polyphenols
Elderberry (*Sambucus nigra* L.)	*Adoxaceae*	nd	78.00 ± 3.12 ^e^	43.37 ± 0.05 ^h^	188.04 ± 3.49 ^l^	63.05 ± 1.55 ^h^	372.47 ± 8.21 ^k^
Arnica (*Arnica* L.)	*Asteraceae*	nd	214.86 ± 7.73 ^c^	65.29 ± 3.14 ^f^	1397.16 ± 31.07 ^b^	22.35 ± 0.35 ^i^	1699.65 ± 42.29 ^e^
Chamomile (*Matricaria* L.)	*Asteraceae*	nd	189.82 ± 10.68 ^c^	112.69 ± 9.17 ^d^	405.50 ± 16.69 ^h^	21.16 ± 2.33 ^i^	729.17 ± 38.87 ^j^
Cornflower (*Centaurea cyanus* L.)	*Asteraceae*	1.68 ^ǂ^ ± 0.00 ^e^	47.85 ± 1.06 ^f^	22.03 ± 0.32 ^i^	131.56 ± 3.09 ^m^	81.16 ± 3.88 ^g^	284.27 ± 8.35 ^l^
Daisy (*Bellis perennis* L.)	*Asteraceae*	nd	350.40 ± 9.47 ^a^	74.81 ± 3.50 ^e^	773.66 ± 5.85 ^e^	200.32 ± 8.45 ^f^	1399.19 ± 27.27 ^f^
Marigold (*Calendula officinalis* L.)	*Asteraceae*	nd	14.90 ± 0.32 ^h^	336.66 ± 14.33 ^b^	1794.54 ± 15.05 ^a^	nd	2146.10 ± 29.70 ^d^
Acacia (*Acacia* Mill.)	*Fabaceae*	nd	35.12 ± 2.04 ^g^	330.18 ± 17.49 ^b^	427.97 ± 5.11 ^h^	446.91 ± 34.00 ^e^	1240.19 ± 58.64 ^g^
Kidney vetch (*Anthyllis vulneraria* L.)	*Fabaceae*	nd	1.47 ± 0.02 ^k^	51.87 ± 3.00 ^g^	202.21 ± 5.55 ^k^	2690.15 ± 15.77 ^c^	2945.70 ± 24.34 ^c^
Lavender (*Lavandula* L.)	*Lamiaceae*	8.37 ± 0.19 ^d^	201.11 ± 8.29 ^c^	18.68 ± 1.08 ^i^	989.37 ± 11.54 ^c^	nd	1217.53 ± 21.10 ^g^
White dead-nettle (*Lamium album* L.)	*Lamiaceae*	247.56 ± 5.13 ^b^	151.54 ± 5.55 ^d^	47.20 ± 1.60 ^g^	542.59 ± 14.67 ^g^	nd	988.88 ± 26.95 ^h^
Black hollyhock (*Malvae arboreae* L.)	*Malvaceae*	286.14 ± 7.02 ^a^	45.91 ± 2.06 ^f^	131.78 ± 16.66 ^d^	544.40 ± 6.04 ^g^	nd	1008.23 ± 31.78 ^h^
Linden blossom (*Tilia cordata* Mill.)	*Malvaceae*	nd	3.66 ± 0.08 ^j^	10.76 ± 0.48 ^j^	275.83 ± 1.01 ^j^	4211.73 ± 81.88 ^b^	4501.98 ± 83.45 ^b^
Mallow (*Malva* L.)	*Malvaceae*	13.38 ± 0.21 ^c^	161.53 ± 8.60 ^d^	72.00 ± 2.44 ^e^	654.73 ± 12.00 ^f^	nd	901.65 ± 23.25 ^i^
Primrose (*Primula* L.)	*Primulaceae*	nd	9.00 ± 0.06 ^i^	849.96 ± 12.06 ^a^	927.16 ± 18.21 ^d^	2537.69 ± 78.19 ^d^	4323.80 ± 108.52 ^b^
Hawthorn (*Crataegus* L.)	*Rosaceae*	nd	245.53 ± 11.94 ^b^	193.71 ± 6.05 ^c^	1384.45 ± 20.11 ^b^	5284.04 ± 62.01 ^a^	7107.73 ± 100.11 ^a^
Mullein (*Verbascum* L.)	*Scrophulariaceae*	0.74 ± 0.03 ^f^	31.71 ± 2.40 ^g^	20.16 ± 2.96 ^i^	293.14 ± 4.93 ^i^	nd	355.76 ± 10.32 ^k^

nd—not detected; ^ǂ^ values are means of three repetitions; mean values followed by different letters (^a–l^) are statistically different at *p* < 0.05.

**Table 2:** Content of triterpenoids and tetraterpenoids (mg/100 g dm) in 16 selected edible flowers.

Kind of Edible Flowers	Carotenoids	Chlorophylls	Triterpenoids
Elderberry	33.33 ^ǂ^ ± 2.11 ^m^	2.14 ± 0.15 ^i^	0.03 ± 0.00 ^i^
Arnica	558.51 ± 8.04 ^b^	38.42 ± 1.94 ^b^	0.56 ± 0.03 ^b^
Chamomile	51.49 ± 1.00 ^j^	7.25 ± 0.15 ^g^	0.05 ± 0.01 ^h^
Cornflower	30.00 ± 1.00 ^m^	nd	0.03 ± 0.01 ^i^
Daisy	174.22 ± 1.69 ^f^	14.21 ± 0.06 ^e^	0.17 ± 0.02 ^e^
Marigold	721.49 ± 9.05 ^a^	nd	0.72 ± 0.00 ^a^
Acacia	31.33 ± 0.94 ^m^	5.48 ± 0.01 ^h^	0.03 ± 0.01 ^i^
Kidney vetch	74.43 ± 8.36 ^i^	9.23 ± 0.01 ^f^	0.07 ± 0.00 ^g^
Lavender	380.61 ± 5.33 ^d^	59.45 ± 0.96 ^a^	0.38 ± 0.02 ^d^
White dead-nettle	48.15 ± 0.03 ^k^	nd	0.05 ± 0.01 ^h^
Black hollyhock	40.29 ± 1.27 ^l^	nd	0.04 ± 0.01 ^hi^
Linden blossom	130.70 ± 2.11 ^g^	19.70 ± 0.06 ^d^	0.13 ± 0.01 ^f^
Mallow	203.91 ± 2.55 ^e^	28.84 ± 0.02 ^c^	0.20 ± 0.02 ^e^
Primrose	34.10 ± 2.03 ^m^	nd	0.03 ± 0.00 ^i^
Hawthorn	472.60 ± 9.65 ^c^	17.07 ± 2.83 ^d^	0.47 ± 0.02 ^c^
Mullein	115.27 ± 3.21 ^h^	nd	0.12 ± 0.00 ^f^

nd—not detected; ^ǂ^ values are means of three repetitions; mean values followed by different letters (^a–l^) are statistically different at *p* ≤ 0.05.

**Table 3:** Enzyme of α-amylase. α-glucosidase, acetylcholinesterase and butyrylcholinesterase inhibitory activities in 16 selected edible flowers.

Kind of Edible Flowers	Enzyme Inhibition IC_50_ (mg of Dried Flowers)			
	Anti-Hyperglycemic Activities		Anti-Aging Activities	
	α-amylase	α-glucosidase	AChE	BuChE
Elderberry	7.17 ± 0.08 ^d^	>100.00 ^g^	86.36 ± 1.05 ^g^	246.53 ± 4.07 ^i^
Arnica	4.06 ^ǂ^ ± 0.04 ^b^	13.99 ± 0.11 ^b^	47.31 ± 0.55 ^d^	122.02 ± 1.55 ^e^
Chamomile	6.37 ± 0.26 ^c^	54.23 ± 0.72 ^e^	40.52 ± 0.98 ^c^	96.14 ± 2.11 ^d^
Cornflower	8.77 ± 0.31 ^g^	>100.00 ^g^	31.64 ± 0.07 ^a^	51.81 ± 0.05 ^a^
Daisy	8.48 ± 0.07 ^f^	49.62 ± 0.01 ^d^	107.79 ± 2.88 ^j^	136.33 ± 2.02 ^f^
Marigold	8.96 ± 0.09 ^g^	>100 g	92.28 ± 1.15 ^h^	135.39 ± 0.57 ^f^
Acacia	8.89 ± 0.21 ^g^	45.46 ± 0.05 ^c^	270.60 ± 4.33	332.05 ± 7.18 ^j^
Kidney vetch	3.50 ± 0.09 ^a^	>100.00 ^g^	33.73 ± 1.01 ^b^	82.93 ± 1.11 ^c^
Lavender	8.71 ± 0.19 ^g^	>100.00 ^g^	97.96 ± 1.69 ^i^	70.69 ± 0.95 ^b^
White dead-nettle	8.21 ± 0.26 ^f^	>100.00 ^g^	75.11 ± 0.95 ^f^	257.10 ± 4.14 ^i^
Black hollyhock	7.15 ± 0.09 ^d^	>100.00 ^g^	71.59 ± 1.22 ^e^	198.21 ± 3.61 ^h^
Linden blossom	8.24 ± 0.14 ^f^	10.79 ± 0.38 ^a^	73.76 ± 2.08 ^e^	199.91 ± 5.09 ^h^
Mallow	7.57 ± 0.09 ^e^	76.75 ± 0.82 ^f^	191.63 ± 2.17 ^k^	379.31 ± 8.55 ^k^
Primrose	11.69 ± 0.11 ^i^	10.18 ± 0.23 ^a^	86.84 ± 1.11 ^g^	94.20 ± 1.11 ^d^
Hawthorn	10.71 ± 0.11 ^h^	10.72 ± 0.43 ^a^	69.59 ± 1.12 ^e^	132.70 ± 2.12 ^f^
Mullein	8.99 ± 0.21 ^g^	76.57 ± 1.12 ^f^	102.90 ± 3.76 ^i^	169.08 ± 4.12 ^g^

nd—not detected; ^ǂ^ values are means of three repetitions; mean values followed by different letters (^a–k^) are statistically different at *p* ≤ 0.05.
